# The Impact of High CMV Viral Load and Refractory CMV Infection on Pediatric HSCT Recipients with Underlying Non-Malignant Disorder

**DOI:** 10.3390/jcm11175187

**Published:** 2022-09-01

**Authors:** Zofia Szmit, Jowita Frączkiewicz, Małgorzata Salamonowicz-Bodzioch, Anna Król, Marek Ussowicz, Monika Mielcarek-Siedziuk, Karolina Liszka, Paweł Marschollek, Ewa Gorczyńska, Krzysztof Kałwak

**Affiliations:** Department of Pediatric Hematology/Oncology and BMT, Wroclaw Medical University, Supraregional Center of Pediatric Oncology “Cape of Hope”, 50-556 Wrocław, Poland

**Keywords:** hematopoietic stem cell transplantation, infectious complications, cytomegalovirus, pediatric

## Abstract

Hematopoietic stem cell transplantation (HSCT) is a curative therapy for an increasing number of nonmalignant indications. Its use is restricted by severe transplant-related complications, including CMV infection; despite various prophylactic and therapeutic strategies, CMV reactivation has remarkable morbidity and mortality. The analysis included 94 children with nonmalignant disorder who underwent allogeneic HSCT in the Department of Pediatric Hematology, Oncology, and Bone Marrow Transplantation in Wrocław during years 2016–2020. Twenty-seven (29%) children presented with CMV infection, including ten (10/27; 37%) with high level CMV viremia (10,000 copies/mL). Six patients experienced subsequent CMV reactivation. The first-line ganciclovir-based (GCV) treatment was insufficient in 40% (11/27) of children. Overall survival (OS) was significantly lower in children with high CMV viremia compared to those with low levels/no CMV [1yrOS High CMV = 0.80 (95% CI 0.41–0.95) vs. 1yrOS others = 0.96 (95% CI 0.89–0.99)]. Similarly, patients with resistant and recurrent infections had greater risk of death. CMV reactivation at any level relevantly prolonged the hospital stay. CMV reactivation with high viremia load and resistant/recurrent CMV infections lead to a significant decrease in OS in children with nonmalignant disorders treated with HSCT. Our data proves there is an urgent need to introduce an effective anti-CMV prophylaxis in this cohort of patients.

## 1. Introduction

Allogeneic hematopoietic stem cell transplantation (HSCT) has proved to be an effective treatment method for various diseases. For years, hematological malignancies have been a leading indication for HSCT. Furthermore, HSCT is a curative approach for a constantly increasing number of non-malignant disorders [1,2]. Despite advances made over the decades, it remains a high-risk procedure, contributing significantly to morbidity and mortality [3]. HSCT is associated with life-threatening toxicities leading to direct tissue damage, while long-lasting immunosuppression and slow immune recovery make the recipients susceptible to infections [4,5]. Those severe post-transplant complications are the most relevant obstacle to the broader use of HSCT, particularly in the non-malignant transplant setting.

Cytomegalovirus (CMV), a highly prevalent herpesvirus, comprises the main cause of viral complications post-HSCT, correlating with significantly higher mortality. Although the implementation of pre-emptive therapy (PET) has led to a noticeable reduction in early CMV disease after transplantation, toxicity associated with the available antiviral agents remains challenging. Particularly, the ganciclovir-based approach might correlate with a higher prevalence of invasive fungal infections and delayed immune reconstitution, increasing the risk for late CMV disease [6,7,8,9]. Moreover, CMV with gene mutation towards various antiviral drugs remains a great problem to resolve [8,10,11].

Repeated CMV DNA monitoring is a part of standard post-HSCT care, at least until day 100. It allows for early detection of patients at risk for CMV disease, who requires implementation of pre-emptive therapy, while it may spare others from the toxicity of universal prophylaxis [8,12,13,14]. Nevertheless, this landscape is changing due to the broader use of letermovir, a novel antiviral prophylactic agent with a favorable safety profile. However, its use is limited to adult HSCT recipients; at present, it is not registered in pediatrics [15].

Although CMV DNA viral load testing by qPCR is increasingly used to guide the pre-emptive therapy, the data considering the correlation between specific viral load thresholds and clinical outcomes are sparse [16].

This single-center retrospective study aimed to evaluate the incidence and outcome of CMV reactivation in non-malignant pediatric HSCT recipients, including an analysis of the probable correlation between CMV viral load and survival.

## 2. Study Design

The study incorporated all children and adolescents with a non-malignant disorder who underwent allogeneic HSCT in the Department of Pediatric Hematology, Oncology, and Bone Marrow Transplantation during the years 2015–2020. Therefore, 94 individuals (60 males/34 females) aged from 2 months to 19 years (median 3.8 yrs) were enrolled into the study. The post-transplant course was reviewed from day 0 (the day of donor cell infusion) to the last documented clinical evaluation (minimum of one year). Apart from directly CMV-related data, the epidemiological and demographic factors, including donor-recipient serostatus, were assessed in terms of general outcome. Therefore, several outcome measures, including present status, cause of mortality, length of hospital stay, and acute GVHD, were captured. Detailed indications for HSCT in the study cohort are presented in Table 1.

Grading and staging of aGvHD were performed using pediatric-specific criteria published by Jacobsohn et al. For our research, only grades II–IV were considered.

### 2.1. CMV Diagnosis and Treatment

All patients received common antiviral prophylaxis which consisted of acyclovir at the dose of 10–15 mg/kg/day starting from day 10 of transplant. This treatment was either discontinued on immune reconstitution or substituted for another antiviral whenever CMV infection was confirmed.

Viral load was assessed in serum by real-time quantitative PCR on a weekly basis starting from the first week post-HSCT. Identified levels above 500 copies/mL were considered as CMV reactivation [9,12]. High level reactivation was defined as >10,000 copies/mL.

Individuals with CMV reactivation received pre-emptive treatment consisting of ganciclovir (GCV) at the dose of 5mg/kg BID. Those who presented with increasing CMV viral load or did not achieve resolution of CMV viremia were considered GCV-resistant and required modification of the treatment. CMV refractoriness was defined as documented failure to achieve >1 log10 decrease in DNA level after two weeks of treatment. The most common second-line agent was foscarnet (180 mg/kg daily) [7,12,17].

Recurrent infection was defined as new detection of CMV in patients who had previously presented and recovered from CMV infection. Only patients with a documented period of at least four weeks of non-detectable CMV viremia were considered as experiencing multiple reactivations [6].

### 2.2. Study End-Points

The primary endpoints of the study were overall survival (OS) and event-free survival (EFS). The OS was defined as the duration of survival from HSCT until the time of death from any cause, and data on the patients still alive were censored at the date of last follow-up. In the EFS analysis, primary graft failure, graft rejection, and death from any cause was considered an event, and the patients were censored at the last follow-up.

The secondary outcome of the study was the length of hospital stay during the first 100 days post-transplant as an indirect measure of the early post-HSCT quality of life.

### 2.3. Statistical Methods

The elementary characteristics in the study cohort were expressed as percentages for the discrete variables and median values for continuous variables. Utilizing either chi-squared or Fisher exact test for discrete variables and the Wilcoxon nonparametric rank sum test for continuous data, we first compared the baseline characteristics of patients with and without CMV reactivation. The *p* values lower than 0.05 were considered significant. Thereafter, we performed survival analysis using Kaplan–Meier estimation. We have applied the landmark analysis with the landmark time of 100 days, where appropriate. Finally, we used Cox proportional hazard model to evaluate the factors influencing patients’ survival. Univariate Cox models were estimated for all patients’ characteristics. The starting point for multivariate Cox models were only those variables from the univariate analysis which were significant at the level of 0.1. The final multivariate model consists solely of highly significant variables (*p* values lower than 0.05)

All statistical analyses were performed using R statistical software ver. 4.1.1. (The R Foundation for Statistical Computing, Auckland, CA, USA)

## 3. Results

In the analyzed time, 29% (27/94) of HSCT recipients presented with CMV reactivation (10 females/17 males). Among those, 23 (85%) were CMV seropositive, and 4 (15%) were CMV seronegative prior transplantation. In the entire study cohort, 64 (68%) patients developed grade II–IV aGvHD, including 11 patients in the CMV reactivation group. However, the statistical comparison of patients with and without CMV reactivation failed to confirm any correlation between CMV viremia and aGvHD (Table 2). Among patients presenting with CMV infection, 37% (10/27) developed high CMV viremia levels. The median detected viral load was 1770 copies/mL in the whole cohort and 24,050 copies/mL in the high CMV viremia subgroup. The median time of CMV reactivation was 27.5 days post-HSCT (range: 24–78 days). One patient developed CMV disease (pneumonia).

The pre-emptive treatment was administered to every patient with detectable CMV viremia (>500 copies/mL threshold). The initial anti-CMV treatment was insufficient in over 40% (11/27) of children. Those who developed GCV resistance received second-line treatment consisting of foscarnet alone (*n* = 6) or with CMV IgG hyperimmunoglobulin preparation (*n* = 5). Three patients received cidofovir; however, it was administered due to either coexisting BKV or EBV infection.

Six patients experienced second CMV reactivation. Only one patient presented with three independent CMV reactivations (Table 3).

In the whole analyzed cohort, 12 patients died. In all cases, death was due to TRM; one patient died due to CMV pneumonia and one due to severe aGvHD with high viral load CMV infection. Two other patients died from invasive aspergillosis.

The study cohort’s one-year and three-year OS was 0.9 and 0.86, respectively.

The OS and EFS were relevantly lower among patients with higher CMV viral load compared to those with either low CMV levels or no reactivation [1yr OS High CMV = 0.80 (95% CI 0.41–0.95) vs. 1yr OS Others = 0.96 (95% CI 0.89–0.99)]. Similarly, children suffering from remittent CMV and experiencing recurrent infection had lower chances for survival [1yr OS recurrent infection = 0.67 (95% CI 0.19–0.90) vs. 1yr OS Others = 0.96 (95% CI 0.89–0.99)].

**Figure 1:**
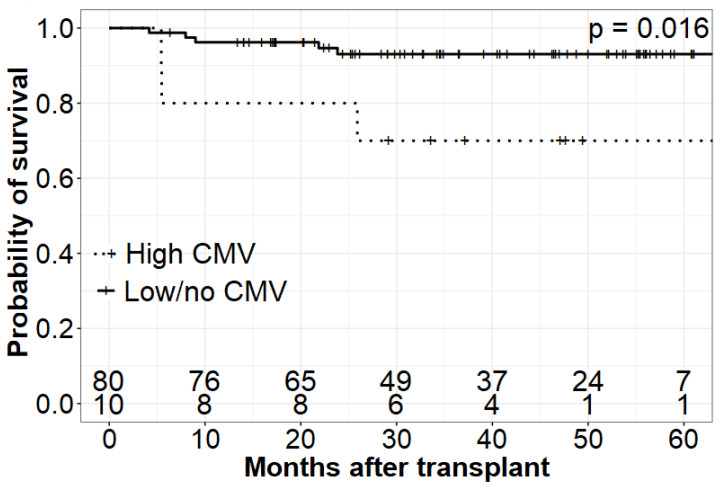
Comparison of OS of patients with high CMV viremia and those with low viremia/no CMV infection.

**Figure 2:**
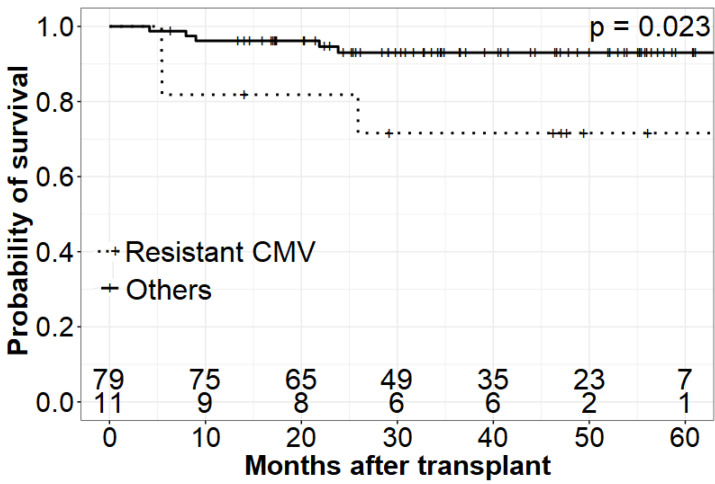
Overall survival of patients with resistant CMV infection.

**Figure 3:**
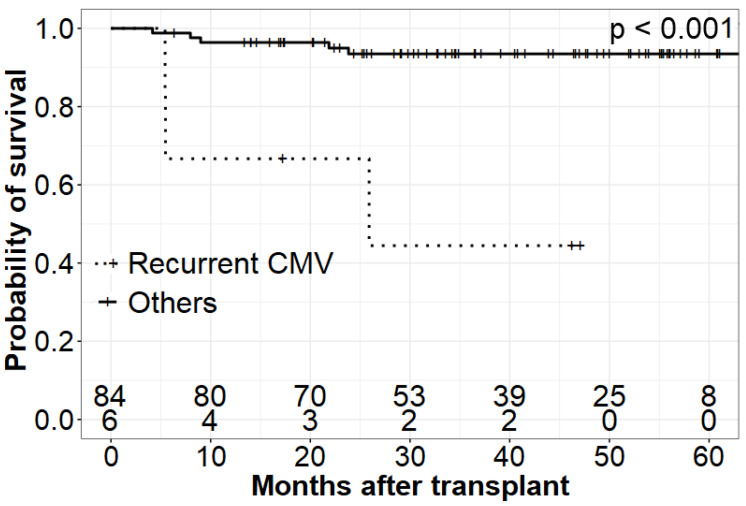
Overall survival of patients with recurrent CMV infection.

Patients experiencing GCV resistance and requiring alternative treatment were at higher risk of death compared to patients with GCV-sensitive CMV and those without CMV reactivation [1yr OS GCV resistant = 0.82 (95% CI 0.45–0.95) vs. 1yr OS Others = 0.96 (95% CI 0.89–0.99)]. A detailed and graphic presentation of survival analysis is demonstrated in Figure 1, Figure 2 and Figure 3 and Table 4.

Apart from the Kaplan–Meier estimation, we performed Cox proportional models to further investigate the factors that may influence survival. A multivariate survival analysis confirmed high CMV viral load as an independent factor worsening survival (Table 5 and Table 6).

Apart from the survival, there was a significant difference in the median length of hospital stay during the first 100 days post-HSCT among those with and without CMV reactivation (median 48 days vs. 37 days; *p* = 0.0003)

## 4. Discussion

Treating non-malignant disorders with HSCT requires a different approach than classic, malignant indications. While we are not battling cancer, careful risk stratification, focusing on post-HSCT complications and adequate prophylaxis, seems essential.

Notably, reducing severe opportunistic infections affecting HSCT recipients might extend the use of HSCT in benign diseases [19,20].

CMV reactivation is one of the most common post-transplant viral infections. Its incidence varies between 12.8% and 60% (encompassing the 27% incidence in our cohort). Such discrepancy between centers arises from different preemptive strategies and institutional CMV viremia threshold defining the CMV reactivation (150–1000 copies/mL). Furthermore, it depends on the latent CMV prevalence in donor population which may vary on sociogeographical status [21,22,23,24,25,26,27]. HSCT recipients with underlying non-malignant disorder hold no advantage in CMV incidence over those with malignancy [26,28].

In our cohort, all patients experiencing CMV reactivation (viremia > 500copies/mL) received standard first-line treatment consisting of GCV, which turned out to be abortive in almost 40%. It resulted in an 11.5% incidence of refractory CMV in the analyzed group. The reported prevalence of refractory CMV varies from 0% to 11.9% in HSCT recipients (from HLA-matched donors); however, it rises to over 14% in high-risk patients from haploidentical donors [29,30,31,32]. The increased risk for refractory CMV in haploidentical HSCT recipients failed to be confirmed in our study. The documented outcome of the rescue therapy for refractory and GCV-resistant severe CMV disease is variable. In our study, patients developing CMV refractoriness had significantly lower survival chances than responders for initial antiviral treatment and those without CMV reactivation. The novel currently investigated agents are offering the potential of effective second/third-line treatment for resistant CMV infection (including maribavir, leflunomide, and virus-specific T-cell therapy). In addition, laboratory testing and genotyping for mutations in UL97 or UL54 might be particularly helpful in choosing the right direct or adjunctive therapy. Such a multivariate approach and prompt identification of those at risk of refractory CMV should relevantly influence the outcome of non-malignant HSCT recipients [10,11,30].

Similar to CMV refractoriness, patients who experienced recurrent CMV infection had a significantly greater chance of death at any cause compared to controls. Recurrent viremia is a well-known risk factor for both CMV disease and post- HSCT mortality [32,33]. In our study, most of the patients experiencing a second episode of CMV viremia were those who presented with high viral load during the first episode. According to published data, the end-treatment viral loads and the kinetics of viremia clearance might be the indicator of progression to CMV disease and general outcome [34,35]. Despite that all but one patient in our study reached the level of non-detectable CMV DNA, higher viral load during treatment seemed to correlate with the CMV recurrence.

Apart from resistant and recurrent CMV, all patients presenting with high CMV levels had significantly lower chances for survival. Numerous studies identified CMV infection as a predictor for increased mortality; however, in a majority of them, CMV was analyzed as a binary event [25,26,36]. Despite that CMV viral load is widely used to guide the initiation of PET, the data about correlation of viral load and clinical endpoints are lacking. Only a few studies are proving a link between higher viral load and development of CMV disease [23,37,38,39]. However, a noticeable percentage of patients in those trials did not receive PET, which currently remains a game-changer in the CMV disease prevalence [40,41].

In our cohort, patients presenting with higher viral load had lower OS and EFS compared to those with lower CMV levels. In addition, our data somehow support the correlation between viral load and the CMV disease as the only case of CMV disease observed in our cohort, which presented with extremely high CMV viremia [34,38].

Apart from the survival, patients who experienced any kind of CMV reactivation had significantly longer accumulated time of hospitalization within the 100 days post-HSCT. To our belief, it may affect both patients’ quality of life in the earliest post-transplant period as well as have adverse impact on economic issues.

Contrary to the literature reports, our data failed to confirm the impact of donor-recipient serostatus on the CMV infection. There is a noticeable tendency towards higher prevalence of CMV infection in the high-risk patients (mainly seropositive recipients), and to the best of our belief, the lack of statistical significance is caused by relatively small patient sample. However, we found a statistically significant correlation between the donor-recipient serostatus and high CMV viral load. All patients suffering from high viral load CMV infection were CMV seropositive prior transplantation (*p* < 0.05)

Numerous trials comparing antiviral prophylactic strategies towards CMV (including acyclovir, ganciclovir, maribavir, and brincidofovir) showed relevant decrease in CMV disease but rather low or no impact on the survival. The only agent, for now, which has been proven to decrease all cause mortality when used as CMV prophylaxis is letermovir. [15,42,43,44]. Aside from a large cohort trial, there are some smaller but most up to date reports delivering real-world evidence of the effectiveness of letermovir in clinical practice [45,46]. Recently, its use became common in adult transplant recipients; however, it is very limited in pediatrics due to its lack of registration in children. Apart from confirmed impact on direct mortality, primary letermovir-based prophylaxis have the capability of decreasing the percentage of refractory and resistant CMV infections as well as the peak CMV viral load [31]. Our data provides relevant evidence that drugs and agents preventing the high CMV viral loads post-transplant might be expected to influence mortality even if they cannot completely prevent the viremia or the need for PET initiation. Furthermore, the presented results support the utility of CMV viremia levels as a surrogate endpoint predicting clinical outcome or treatment response as it is widely used in other viral infection such as HIV and HCV [47].

The study’s major limitations are its retrospective nature and relatively low number of patients. Doubtlessly, there is an urgent need for prospective cohort studies regarding CMV and its prophylaxis among pediatric transplant recipients, particularly those with non-malignant diseases.

To conclude, CMV reactivation with high viremia load and resistant/recurrent CMV infections lead to a significant reduction in overall survival in children with non-malignant disorders treated with HSCT. Our results strongly support the necessity to introduce an effective anti-CMV prophylaxis in this cohort of patients. Letermovir offers the potential to decrease the rate of CMV infection along with reduction in viremia levels and thus improve the OS and patients’ quality of life.

## Figures and Tables

**Table 1:** Indications for HSCT in the study cohort.

Diagnosis		Total *n* = 94
**Anemias** ***n*** **= 38**	Severe aplastic anemia	32
	Fanconi anemia	3
	Other	3
**Immunodeficiency** ***n*** **= 37**	Wiskott-Aldrich syndrome	13
	SCID	8
	HLH	3
	Nijmegen syndrome	2
	Other	11
**Metabolic disorders** ***n*** **= 19**	Metachromatic Leukodystrophy	5
	X-ALD	3
	Hurler syndrome	3
	Other	8

Abbreviations: SCID—Severe combined immunodeficiency disorder; HLH—Hemophagocytic lymphohistiocytosis; X-ALD—X-linked adenoleukodystrophy.

**Table 2:** Characteristics of study cohort. Comparison of patients with and without CMV reactivation.

		No CMV Reactivation *n* (%)	CMV Reactivation *n* (%)	*p* Value
**Total *n* = 94**		67	27	
**Sex**	Female	24 (35.8)	10 (37)	0.912
	Male	43 (64.2)	17 (63)	
**Diagnosis**	Anemias	26 (38.8)	12 (44.4)	0.433
	Immunodeficiency	29 (43.3)	8 (29.6)	
	Metabolic disorders	12 (17.9)	7 (25.9)	
**Type of donor**	MMRD	3 (4.5)	1 (3.7)	1
	MSD	14 (20.9)	6 (22.2)	
	MUD	50 (74.6)	20 (74.1)	
**Stem cell source**	BM	13 (19.4)	6 (22.2)	0.907
	CBU	3 (4.5)	1 (3.7)	
	PBSC	51 (76.1)	20 (74.1)	
**Conditioning regimen**	MAC	15 (22.4)	6 (22.2)	0.852
	NMA	31 (46.3)	11 (40.7)	
	RIC	21 (31.3)	10 (37)	
**aGvHD**	aGvHD 2–4	23 (34.3)	7 (25.9)	0.429
	No aGvHD	44 (65.7)	20 (74.1)	
**GvHD prophylaxis**	With ATG	62 (92.5)	27 (100)	0.317
	Without ATG	5 (7.5)	0 (0)	
**Donor-recipient** **serostatus**	R+/D+	26 (38.8)	17 (63)	0.284
	R+/D−	21 (31.3)	6 (22.2)	
	R−/D+	10 (14.9)	3 (11.1)	
	R−/D−	9 (13.4)	1 (3.7)	
**Donor-recipient** **CMV risk status**	R+/D+ or R+/D− (high risk)	47 (71.2)	23 (85.2)	0.156
	R−/D+ or R−/D− (low risk)	19 (28.8)	4 (14.8)	
**Donor-recipient** **CMV risk status**	R−/D− (low risk)	9 (13.6)	1 (3.7)	0.271
	R+/D+ or R+/D− or R−/D+	57 (86.4)	26 (96.3)	

Abbreviations: MMRD—Mismatched related donor; MSD—Matched sibling donor; MUD—Matched unrelated donor; BM—Bone marrow; CBU—Cord blood unit; PBSC—Peripheral blood stem cells; NMA—Non myeloablative conditioning regimen; MAC—Myeloablative conditioning regimen; RIC—Reduced intensity conditioning regimen [18]; ATG—Antithymocyte globulin.

**Table 3:** Details of CMV reactivation in the study cohort.

	No. of Patients with CMV Reactivation *n* = 27
**High level reactivation (>10,000 copies/mL)**	10 (37%)
Median CMV viremia level	Median 1770 copies/mL (range 1075–79,000 copies/mL)
**Recurrent infection**	6 (22%)
Resistant infection	11 (40.7%)
**Time of CMV reactivation diagnosis**	Median 27.5 days post-HSCT (range 24–78 days)

**Table 4:** Analysis of 3-years-EFS.

		3yr Event-Free Survival	*p*
**High CMV viral load**	Yes	0.60 (95% CI 0.25–0.83)	0.017
	No	0.87 (95% CI 0.75–0.92)	
**Resistant infection**	Yes	0.53 (95% CI 0.21–0.77)	0.0004
	No	0.87 (95% CI 0.77–0.93)	
**Recurrent infection**	Yes	0.25 (95% CI 0.01–0.65)	0.0001
	No	0.86 (95% CI 0.76–0.92)	

**Table 5:** Results of univariate Cox analysis of factors influencing survival.

		3yr Overall Survival	
Variable		Hazard Ratio (95% CI)	*p*
**Sex**	Male	0.27 (0.08–0.88)	0.03 *
**Diagnosis**	ID	1.26 (0.33–4.71)	0.73
	IE	1.57 (0.35–7.03)	0.55
**Type of donor**	MSD	0.15 (0.01–2.48)	0.19
	MUD	0.45 (0.06–3.5)	0.44
**Stem cell source**	CBT	5.03 (0.07–35.8)	0.11
	PBSCT	0.99 (0.21–4.68)	0.99
**Conditioning regimen**	NMA	1.47 (0.29–7.31)	0.63
	RTC	1.80 (0.33–9.84)	0.49
**aGvHD**	aGvHD 2–4	3.04 (0.96–9.57)	0.05 *
**GvHD prophylaxis**	With ATG	0.42 (0.06–3.28)	0.41
**Donor-recipient** **serostatus**	R+/D−	0.83 (0.21–3.33)	0.80
	R−/D+	0.56 (0.07–4.66)	0.59
	R−/D−	1.45 (0.29–7.18)	0.65
**Donor-recipient** **Risk status** **(Ver 1.)**	R−/D+ or R−/D+ (low risk)	1.01 (0.27–3.74)	0.98
**Donor-recipient** **Risk status** **(Ver 2.)**	R−/D− (low risk)	1.65 (0.36–7.53)	0.52
**High CMV**	Yes	2.73 (0.74–10.10)	0.09 *

**Table 6:** Results of Cox analysis of factors influencing survival.

		3yr Overall Survival	
Variable		Hazard Ratio (95% CI)	*p*
**aGvHD**	Yes	1	
	No	0.26 (0.08–0.85)	0.03
**High CMV**	No	1	
	yes	4.10 (1.04–16.14)	0.04

## Data Availability

All data generated or analysed during this study are included in this published article.
